# Acute Palliative Physical Therapy Services for a Patient With Metastatic Rectal Cancer and Subsequent Spinal Cord Compression

**DOI:** 10.7759/cureus.17691

**Published:** 2021-09-03

**Authors:** Meghan K Huber, Christopher M Wilson, Nathan Y Li

**Affiliations:** 1 Physical Therapy, Mayo Clinic, Rochester, USA; 2 Rehabilitation Services, Beaumont Health, Troy, USA; 3 Physical Therapy, Oakland University, Rochester, USA; 4 Pharmaceutical Sciences, University of Michigan, Ann Arbor, USA

**Keywords:** physical therapy rehabilitation, metastatic colo-rectal cancer, palliative care, inpatient rehabilitation, acute care

## Abstract

Colorectal cancer is the third most common cause of cancer-related deaths with approximately 40%-50% of people diagnosed experiencing subsequent metastases. Surgery is the only curative treatment for colorectal cancer, although chemotherapy and radiation are often used neoadjuvantly or adjuvantly to decrease recurrence rates and improve survival. Many individuals experience adverse effects and physical impairments secondary to extensive medical treatment. Therefore, the purpose of this case is to signify the important role of physical therapy in the continuum of care of a patient diagnosed with metastatic rectal cancer and subsequent spinal cord compression. The patient was a 70-year-old male admitted to the hospital for lower extremity (LE) numbness and weakness secondary to metastatic rectal cancer. Seventeen months prior to hospitalization, he was diagnosed with rectal cancer and underwent neoadjuvant chemotherapy and radiation followed by laparoscopic abdominoperineal resection with posterior prostatectomy en bloc with a colostomy. Adjuvant chemotherapy included FOLFIRI. While hospitalized, the patient experienced spinal cord compression secondary to metastasis and elected decompressive laminectomy with discectomy for palliation. Due to the poor prognosis of metastatic rectal cancer, the patient’s functional mobility and independence declined throughout hospitalization. The patient was able to achieve one of two personal goals; he was able to tolerate sitting in an upright position for his daughter’s wedding but unfortunately did not return home prior to expiration. Although the patient suffered eventual mortality, consistent physical therapy allowed him to achieve a major life goal, serving as an important motivator and improved quality of life (QoL) even in end-of-life conditions. Unfortunately, physical therapy services are often overlooked and under-utilized in patients with terminal conditions receiving palliative care, despite the growing body of literature supporting the benefits. By utilizing *rehabilitation in reverse* as well as *skilled maintenance*, physical therapy assists in maintaining mobility and achieving personal goals of individuals with terminal cancer, thus improving QoL even with a poor prognosis.

## Introduction

Colorectal cancer (CRC) is the third most common cause of cancer death in both men and women in the United States [1]. There were approximately 147,950 new cases of CRC in the United States in 2020, including 104,610 cases of colon cancer and 43,340 cases of rectal cancer [2] with approximately 40%-50% eventually developing metastases [1].

Despite the growing incidence, improvements in early detection screenings and treatment possibilities have decreased mortality from CRC in developed nations [3]. The five-year survival rate for those with localized disease is approximately 90% but decreases to 14% for those diagnosed with metastatic disease [1]. Rectal cancer is diagnosed at a localized stage more often than colon cancer, so the five-year relative survival is slightly higher for rectal cancer (67%) as compared to colon cancer (64%) [4].

CRC typically begins as a polyp (noncancerous adenoma) that develops on the inner epithelial lining of the colon or rectum and grows slowly over a period of 10 to 20 years prior to becoming dysplastic [4,5]. Although all adenomas have the potential to become cancerous, fewer than 10% are estimated to progress to invasive cancer [4].

Most patients with rectal adenocarcinoma are diagnosed by colonoscopy after experiencing abnormal bleeding. Currently, surgery is the only curative treatment for rectal cancer and must include resection of the tumor with negative margins [6]. For individuals with metastatic or unresectable disease, surgery may be completed for palliation of pain, obstruction, or bleeding and followed up with a regimen of chemotherapy [7].

Patients with terminal cancer typically experience a significant decline in functional mobility and independence before, during, and following chemotherapy, radiation, and/or surgery. Physical impairments result from multiple factors including deconditioning, muscle fatigue, sarcopenia from direct tumor effects, malnutrition, depression, adverse effects from treatment, bowel and bladder dysfunction, intractable pain, thromboembolic disease, neurologic dysfunction, and other comorbidities [8-10]. These factors contribute to a decline in independent mobility and increased dependence on others, resulting in a significant impact on the patient’s quality of life (QoL) [9-12]. Rehabilitation can assist to restore or maintain function as well as slow down functional decline through skilled interventions including strengthening, ambulation, range of motion (ROM), and pain relief [13].

In order to obtain optimal improvement in functional mobility in individuals with an oncologic diagnosis, Dietz has described four different classifications of cancer rehabilitation, which include preventative, restorative, supportive, and palliative rehabilitation [14]. Each classification has a different goal and is utilized at different periods throughout the cancer journey. Dietz describes supportive physical therapy services as those interventions that provide techniques to improve independent mobility, keeping in mind that a full recovery may not be possible secondary to disease progression and trajectory.

Briggs has constructed further models of physical therapy services in the palliative spectrum which include *rehabilitation light*, *rehabilitation in reverse*, *case management*, *skilled maintenance*, and* supportive care* [15]. *Skilled maintenance* therapy is utilized when a patient is unable to complete an activity independently or with the assistance of a trained family member, thus requiring the skills of a licensed physical therapist [15]. With the inevitable functional decline and dependence present with a terminal diagnosis, the implementation of *rehabilitation in reverse* is of the utmost importance. *Rehabilitation in reverse* is applied to previously independent individuals who are diagnosed and enduring the terminal diagnosis, requiring increased physical assist from their support system and increased reliance on durable medical equipment.

Given the prevalence of CRC, this case was chosen to reflect and review the benefit of rehabilitation services provided concurrently with changing medical status and functional decline in terminal diagnoses. Historically, physical therapy in this context has been severely underutilized, as only 1%-2% of those with physical impairments secondary to cancer diagnosis seek treatment [16]. While many physical therapists focus on improving an individual’s strength and mobility to achieve independence, it is the role of a physical therapist in the oncologic specialty to assist in a safe deterioration experienced with the terminal diagnosis while striving to maintain the optimal functional independence for as long as possible. Thus, the purpose of this case is to describe the clinical decision-making and outcomes of physical therapy during hospitalization for a patient diagnosed with metastatic rectal cancer and subsequent spinal cord compression.

## Case presentation

Patient history and systems review

**Figure 1:**
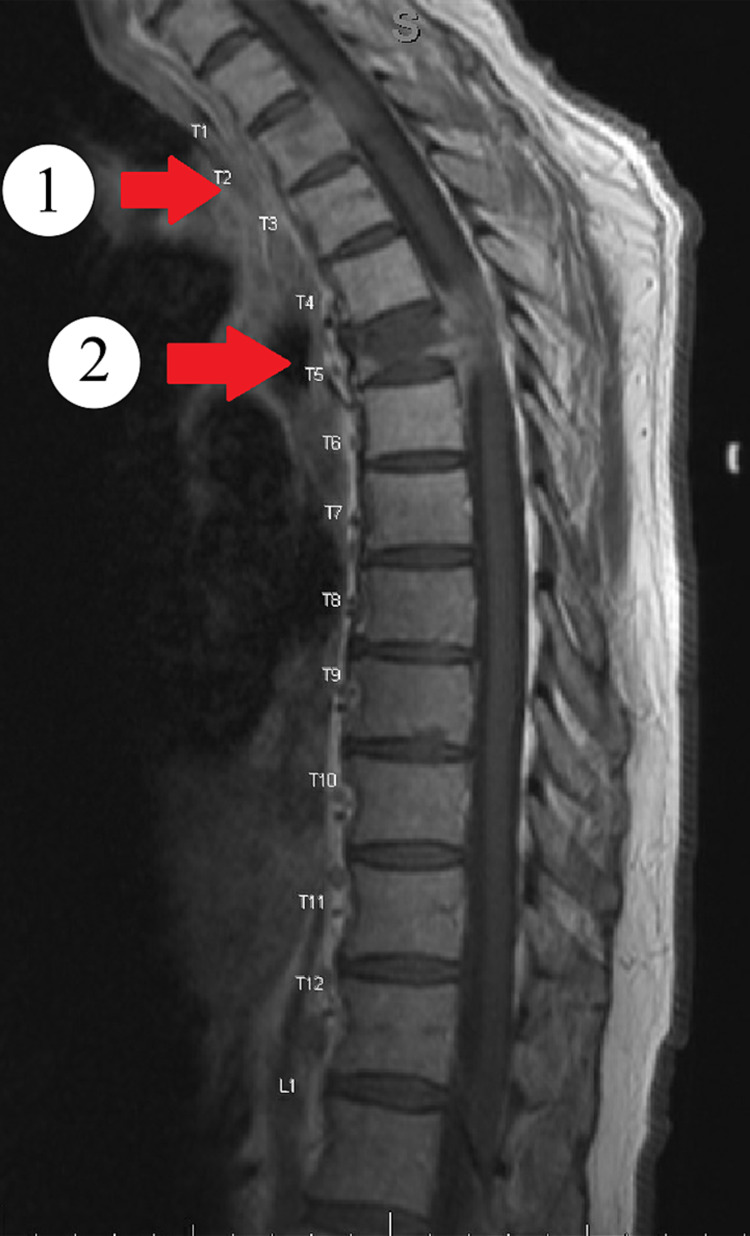
T1-weighted MRI image of the thoracic spine T1-weighted MRI image of the thoracic spine showing (1) an increasing retropulsion and epidural extraosseous metastasis causing moderate canal stenosis and cord compression. Increase in size of osseous metastatic lesion of the T2 vertebral body and left pedicle extending into the left neural foramina causing moderate left neural foraminal stenosis and (2) an increasing moderate to severe pathologic compression fracture deformity of T5 with approximately 75% height loss.

Informed consent was obtained, and the patient agreed to participate in this case report. The patient was a 70-year-old English-speaking, Caucasian male of Middle Eastern descent who was diagnosed with stage IIIb rectal adenocarcinoma 17 months prior to this hospitalization. During that time, cancer metastasized to the thoracic spine, liver, lungs, and left iliopsoas muscle. The patient presented to the emergency center (EC) with a chief complaint of numbness and weakness in his bilateral lower extremities (LEs), although with more symptoms in the left LE. He received palliative radiation to the spine prior to this date and was referred to the EC to rule out cauda equina syndrome. In the EC, a magnetic resonance image (MRI) was completed which indicated a T5 compression fracture resulting in 75% loss of vertebral height with a retropulsed fragment, causing stenosis and cord compression (Figure 1).

Per imaging results, there were no indications of current cauda equina syndrome. The patient was then admitted to the hospital.

**Figure 2:**
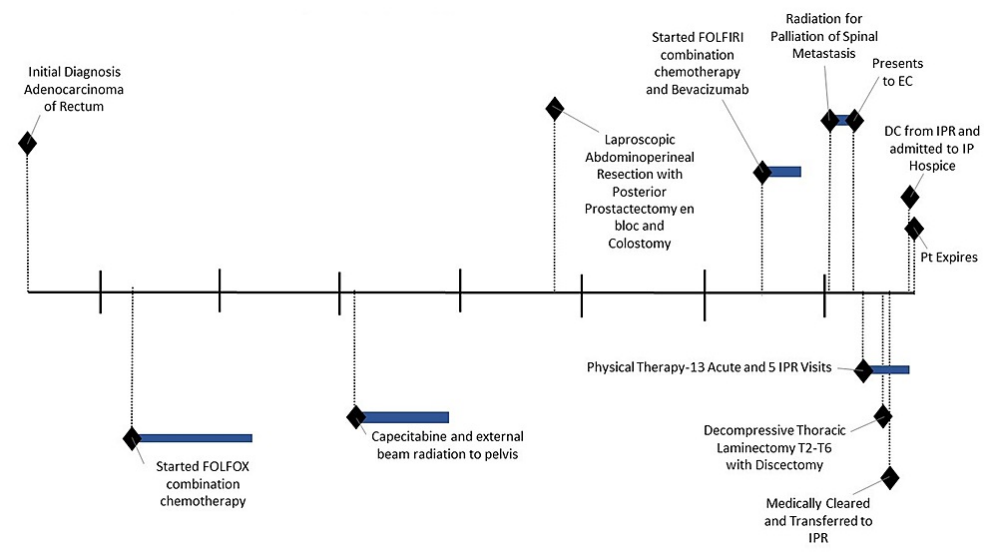
Timeline of events IPR: inpatient rehabilitation; EC: emergency center; DC: discharge; IP: inpatient

The patient had a significant oncologic history which is reviewed in Figure 2. Seventeen months prior to his hospital admission, the patient experienced changes in bowel habits which warranted a colonoscopy. Per colonoscopy and further workup, the patient was clinically diagnosed with cT3N2 invasive adenocarcinoma of the rectum. He did not undergo genetic counseling or testing upon diagnosis.

Less than a month after initial diagnosis, the patient was started on a neoadjuvant chemotherapy regimen of folinic acid (leucovorin), fluorouracil (5-FU), and oxaliplatin (Eloxatin) (FOLFOX), but only completed six cycles due to new-onset and worsening neuropathy. His chemotherapy regimen was switched to capecitabine (Xeloda) while concurrently receiving external beam radiation to the pelvis, rectum, and lymph nodes. The total radiation dose was 5400 cGy which finished approximately six months after initial diagnosis. Two months following the completion of radiation and capecitabine, he underwent a laparoscopic abdominoperineal resection with posterior prostatectomy en bloc with a colostomy. He was pathologically restaged at ypT3, N1b during this surgery.

**Figure 3:**
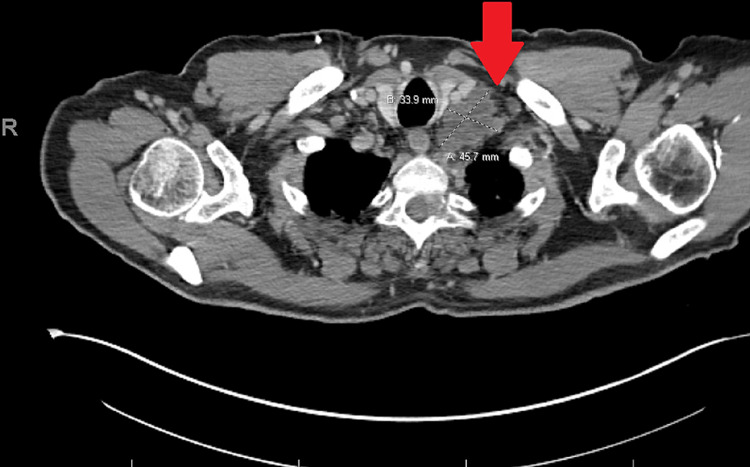
CT image of the pelvis CT image of the pelvis showing conglomeration of pathologic adenopathy and left supraclavicular region (Virchow’s node) measuring 4.6 x 3.4 cm. Additional left supraclavicular node measures 2.4 x 1.6 cm.

Four months after surgery, a computerized tomography (CT) confirmed progression of pelvic and chest metastatic disease (Figure 3), and the patient was started on the combination of leucovorin calcium, 5-fluorouracil, irinotecan, (FOLFIRI), and bevacizumab (Avastin), although stopped after three cycles due to intolerance. With chemotherapy on hold, the patient started palliative radiation to the spinal metastases at T2-3 and T5 for pain control.

The patient had an otherwise insignificant past medical history, although this included colon polyps, gastroesophageal reflux disease (GERD), and ulcerative colitis. He was a former smoker of cigarettes for less than one year approximately 50 years ago. He denied any use of smokeless tobacco, illicit drugs, or alcohol abuse. The patient’s family medical history included one sister with breast cancer, one brother with multiple melanomas, one brother with lung cancer who had a history of smoking, one brother with sarcoma, and one brother with no cancer. A full review of systems is provided in Table 1.

**Table 1:** Review of Systems L: left; LE: lower extremity; R: right; ROM: range of motion; WFL: within functional limits; AROM: active range of motion; UE: upper extremity

Body System or Domain	Clinical Findings
Cardiovascular/Pulmonary	Blood Pressure: 165/86 mmHg; Heart Rate: 90 beats per minute; Peripheral Capillary Oxygen Saturation (SpO_2_): 91% on room air; No signs of tachypnea or labored breathing, voice strong with good projection
Musculoskeletal	170.2 cm 78 kg; BMI 26.93 kg/m^2^; Slight peripheral edema in L LE as compared to R; RROM: WFL bilaterally AROM: moderately limited secondary to weakness; Strength: 4/5 strength bilateral UE, gross 3/5 strength R LE, 2/5 strength L LE
Neuromuscular	Numbness, tingling, decreased light touch and proprioception in bilateral LE although L>R
Integumentary	Integrity: Unimpaired; Skin color/discoloration: no signs of erythema, pallor, cyanosis in bilateral LE
Circulatory	White Blood Cells: 6.4; Red Blood Cells: 4.9; Hemoglobin: 12.7; Hematocrit: 39.4; Mean Corpuscular Volume: 80; Platelets: 255
Communication	Unimpaired
Affect, Cognition, Language, Learning Style	Patient was alert and oriented to person, place, and time; Affect appropriate, motivated and engaged in therapy, many questions throughout sessions; English language

Prior to admission, the patient lived in a two-story home with his wife. There were three stairs to enter the home with no handrails. His bedroom and bathroom were on the first level of the home. He was independent in activities of daily living (ADL) and instrumental activities of daily living (IADL), including driving, and did not utilize an assistive device (AD). He was a retired engineer, although he still assisted in projects as a hobby.

The patient was chosen for a case study due to the rapid onset of physical impairments resulting from metastatic lesions of rectal cancer to the thoracic vertebrae with subsequent spinal cord compression treated with elective surgical intervention.

Examination tests and measures

The physical therapy consult was received and completed four days after admission to the oncology unit. Acute physical therapy evaluation included manual muscle testing (MMT), gross ROM, sensation, and an assessment of functional mobility including bed mobility, transfers, and ambulation. The 5-time sit-to-stand (5STS) [17] was utilized as well as the Activity Measure of Post-Acute Care (AMPAC) “6 clicks” as outcome measures [18].

LE strength was assessed via MMT using a scale from 0 to 5, where a 5/5 indicates maximal strength, 4/5 indicates the individual is able to tolerate added resistance, 3/5 indicates the individual can complete a full ROM against gravity, 2/5 indicates partial movement with limited gravity interference, 1/5 demonstrates trace muscular activation, and 0/5 indicates no contraction or muscular activation [19]. The excellent validity of MMT is supported by significant correlations between MMT testing and dynamometry as well as significant correlations between MMT scores and performance of functional activities (i.e., gait and transfers) [20]. MMT is 0.80-0.99 reliable, with gravity, eliminated positioning obtaining higher reliability [21].

Examination findings revealed right LE strength grossly 3/5 and left LE strength grossly 2/5. His upper extremity (UE) strength bilaterally was 4/5. The sensation was diminished in bilateral LE as assessed by light touch, though the left LE demonstrated more sensory loss compared to the right LE. The pain was subjectively reported at 6/10 on the numeric pain rating scale, with the patient specifying the T5 location [22].

The patient completed bed mobility with a maximum assist of one. The patient required moderate to maximum assistance to complete a sit-to-stand transfer. Initial standing balance was poor with bilateral UE support on an AD. A walker with two front wheels was utilized to complete a stand pivot transfer from the bed to the wheelchair (WC) with a moderate assist. A walker was utilized during the transfer due to LE instability and poor balance. During the 5STS, the patient was only able to complete four repetitions and required 2 minutes and 30 seconds. Gait and stair mobility were deferred at the initial evaluation due to safety concerns and buckling of bilateral LEs upon standing. AMPAC was completed and the patient scored 9/24.

Clinical impression evaluation, diagnosis, and prognosis

Evaluation: Over 72 hours, the patient experienced a sudden onset of LE weakness, decreased sensation, decreased independence in functional mobility, and an increase in pain resulting in significant limitations in functional mobility, ADLs, and IADLs. The 5STS indicated significant lower limb muscle weakness and difficulty performing transitional movements compared to healthy, age-matched peers’ average of 12.6 seconds [17]. The AMPAC is a functional mobility outcome measure based on observed patient activity or clinical judgment utilized in the acute care setting that can assist in determining optimal discharge disposition. A score of 9/24 indicates an 81% degree of functional impairment [19]. Discharge (DC) recommendation at the time of the evaluation was home with a physical assist, which was determined on the evaluation findings, progression of the disease, rehabilitation potential, familial support, and use of the AMPAC score.

The patient was determined to be appropriate for skilled therapy services as he was significantly below his prior level of function. The initial physical therapy plan of care (POC) was established to address the previously mentioned deficits via therapeutic exercise to improve LE strength, neuromuscular re-education to improve static and dynamic sitting balance and improve trunk control, and therapeutic activity to improve independence with functional mobility and transfers. Family training and education were also included as a significant part of the POC to improve the safe handling skills of the family members to care for him and to achieve the ultimate goal of the DC home.

Diagnosis: The admitting medical diagnosis was back pain, unspecified back location, unspecified back pain laterality, unspecified chronicity (ICD 10 code M54.9). The physical therapy diagnoses included generalized muscular weakness (ICD 10 code M62.81), difficulty in walking not elsewhere classified (ICD 10 code R26.2), and other reduced mobility (ICD 10 code Z74.09) [23].

Prognosis: The patient’s rehabilitation potential of functional recovery through physical therapy was poor secondary to progression of the disease and sudden onset of symptoms as well as an established poor prognosis from the medical team [24,25]. Despite his poor prognosis and extent of disease progression, he was motivated and engaged to participate in physical therapy. The patient’s main goal at the initial evaluation was to be able to attend his daughter’s wedding at the end of the month. Other initial acute inpatient physical therapy goals can be reviewed in Table 2.

**Table 2:** Initial Acute Care Goals

Topic of Goal	Anticipated Performance Level
Bed mobility	Minimal assistance
Sit to and from stand	Minimal assistance
Transfer to chair	Minimal assistance
Ambulation	Rolling walker for 10 feet with moderate assistance
Stair mobility	Dependent
Improve sitting balance	Good
Improve standing balance	Fair+
Home exercise program	Independent
Pain management techniques	Independent

Interventions and POC

Physical therapy services were established at a frequency of 5-7 days a week. The patient was also scheduled for occupational therapy at a frequency of 5-7 days a week, thus close collaboration between nursing and occupational therapy was of the utmost importance given his progressive medical condition. Documentation of treatment sessions and interventions occurred in an electronic medical record. Fatigue levels, rate of perceived exertion, and pain levels were monitored throughout each treatment session, and interventions were modified appropriately.

Multiple consults were completed throughout the hospitalization including orthopedic surgery, internal medicine, radiation oncology, urology, infectious disease, medical oncology and hematology, otolaryngology, pulmonary disease, and physical medicine and rehabilitation. Hospice consult was completed early upon admission, although he did not meet medical instability criteria for inpatient hospice at that time. Following the denial for inpatient hospice services and the patient verbalizing he wanted to get stronger to return home, rehabilitation orders were placed along with orthopedic consults for decompressive laminectomy with discectomy.

The patient’s course of physical therapy was split into three phases. During phase one, the patient participated in seven acute care visits that occurred prior to surgery. Phase two entailed six acute care visits that occurred following surgery. Lastly, phase three included the transfer to inpatient rehabilitation (IPR). See Tables 3-5 for specific therapeutic interventions. In addition, lymphedema wrapping was completed on visit 6 prior to surgery.

**Table 3:** Therapeutic Activity Interventions RW: rolling walker; WC: wheelchair

Therapeutic Activity	Phase 1 (Prior to surgery)							Phase 2 (s/p surgery)						Phase 3 (IPR)				
Visit number	1	2	3	4	5	6	7	8	9	10	11	12	13	1	2	3	4	5
Log rolling	x	x	x		x		x	x	x	x	x	x	x	x	x	x	x	
Supine<>sit	x	x	x		x			x	x	x	x	x	x					
Sit<>stand with RW	x	x	x															
Side steps with RW	x	x	x															
Stand pivot to WC	x	x																
Slide board transfer					x													
Mechanical lift to bariatric chair														x	x	x	x	

**Table 4:** Therapeutic Exercise Interventions TrA: transverse abdominus; LE: lower extremity; DF: dorsiflexion; PF: plantarflexion; PROM: passive range of motion; AP: anterioposteriorly

Therapeutic Exercise	Phase 1 (Prior to surgery)							Phase 2 (s/p surgery)						Phase 3 (IPR)				
Visit number	1	2	3	4	5	6	7	8	9	10	11	12	13	1	2	3	4	5
Ankle pumps	x	x	x			x	x			x								
Glute sets	x					x	x			x								
Quad sets	x					x	x			x								
Supine hip abduction/adduction	x					x	x											
Sit to stands			x															
TrA activation				x								x						
Log rolling using overhead trapeze for core engagement				x														
Log rolling with LE propped on wedge					x													
Heel slides					x	x	x											
Manually resisted DF/PF					x													
PROM of LE in supine									x					x	x	x	x	
Adductor sets with pillow											x							

**Table 5:** Neuromuscular Re-education EOB: edge of bed

Neuromuscular Re-education	Phase 1 (Prior to surgery)							Phase 2 (s/p surgery)						Phase 3 (IPR)				
Visit number	1	2	3	4	5	6	7	8	9	10	11	12	13	1	2	3	4	5
Sitting EOB							x	x	x	x		x	x					
Sitting EOB dynamic reaching							x		x	x		x	x					
Sitting EOB posture correction								x	x	x		x	x					
Sitting EOB weight shifting AP and lateral									x			x	x					
Sitting EOB pressure relief										x		x						
Sitting EOB scooting												x						

Phase 1

The acute care PT interventions focused on proper mechanics of log rolling, supine to/from sit, sit to/from stand, stand pivot transfers using the rolling walker (RW), slide board transfers, LE ROM, and LE strengthening exercises in supine and sitting edge of bed (EOB). Education on transverse abdominus activation and core engagement along with pursed-lip breathing, diaphragmatic breathing, and avoidance of Valsalva maneuver were reviewed prior to mobility for each treatment session. Spine precautions (no bending, lifting, twisting) were reviewed and reinforced with all mobility due to metastatic lesions and in preparation for potential elective surgery. The patient demonstrated good carryover of education with decreased pain during mobility. A significant amount of time was allotted each session to educating the patient and family on the disease process and mobility status as well as prognosis.

The patient had an involved family support system who were present for all treatment sessions. His wife, son-in-law, and daughter were the most involved members in his care and completed most of the hands-on family training. The patient and his family were educated on the role of inpatient physical therapy services and interventions focused on improving independence in functional mobility as well as family training to assist the patient in his goal of returning home.

During this phase, the family was instructed on proper positioning of the patient in bed for pressure relief to prevent injury as well as for pain relief. The therapists demonstrated proper body mechanics and techniques for safe patient handling and transfers, including the use of RW for stand pivot transfer and slide board transfer to WC. When the family was comfortable, they completed hands-on participation with therapist feedback and guidance.

After multiple orthopedic consults and a second opinion, the patient and his family elected to undergo palliative surgery. Palliative care assisted the patient in changing his code status to no cardiopulmonary resuscitation prior to the elective surgery with the patient stating, “If I die in surgery that will be a blessing”. Prior to surgery, new physical therapy orders were placed for lymphedema evaluation. A certified lymphedema therapist introduced decongestive therapy [26], including manual lymph drainage to manage his lymphedema and education regarding proper skincare for wound prevention. He was bandaged once using a compression wrap from the foot to the knee on the L LE for palliation prior to surgery. At the end of this phase, the patient became very nervous about his elective spinal surgery but continued to be engaged and motivated to participate in physical therapy.

Phase 2

The patient underwent a decompressive thoracic laminectomy T2-T6 with discectomy with spinal cord monitoring and no complications. Another re-evaluation was completed on visit 8 following the elective surgery. Post-surgically, the patient was instructed to wear a thoracic-lumbar-sacral orthosis (TLSO) for all out-of-bed mobility. Functional mobility assessment was completed with the patient requiring maximum assistance of two people for bed mobility including log rolling and supine to/from EOB. The patient was unable to maintain sitting balance at EOB independently and required maximum assistance from one caregiver with intermittent assistance from a second person. Out-of-bed mobility inclusive of sit to/from stand or transfer to bedside chair was not appropriate and as a result, a 5STS re-test was not completed. At this visit, post-surgical pain was the biggest barrier to physical therapy. Based on his performance, PT goals were updated and can be reviewed in Table 6.

**Table 6:** Updated goals following thoracic decompressive laminectomy with discectomy

Topic of Goal	Anticipated Performance Level
Bed mobility	Maximum assistance
Sit to and from stand	Maximum assistance
Wheelchair mobility	Moderate assistance
Improve sitting balance	Poor+
Improve pain level to	4/10
Home exercise program	Independent
Pain management techniques	Independent

The second phase of physical therapy focused on bed mobility and supine to/from EOB transfers with emphasis on independent sitting balance, tolerance to upright activity, and breathing techniques. With *skilled maintenance* therapy interventions following surgery, he improved his sitting tolerance from 10 minutes to 20 minutes and was able to support himself independently at EOB with bilateral UE assist. He was able to complete small range lateral and anteroposterior weight shifting in sitting but required bilateral UE assistance. The patient continued to require two-person assistance for bed mobility and supine to/from EOB transfer.

Trace quad activation emerged during this time and facilitation techniques were utilized to improve neuromuscular connection. Facilitatory tapping was completed to the quadricep muscle belly while the patient was instructed to perform an open chain knee extension while sitting EOB [27]. This technique was completed until the patient could not tolerate the further exercise, which was approximately 10 repetitions. Family caregiver education continued verbally, although no hands-on practice occurred because of fear of hurting the patient and his frail state post-surgically. Education regarding autonomic dysreflexia was reviewed with the patient and his family.

AMPAC score at initial evaluation was 9/24 and following surgery was 6/24, although this improved to 8/24 in phase two of therapy. The DC recommendations initially were home with physical assistance. Through the acute care treatments following surgery, discharge recommendations were changed to sub-acute rehabilitation (SAR) placement before the final recommendation of IPR for the short stay family training (SSFT) program.

Following visit 12, the patient’s oncologist along with physical medicine and rehabilitation inquired about traditional IPR placement for the patient. A physical therapy re-evaluation to assess DC recommendations occurred and the patient was to be considered for the IPR SSFT program as he would not be able to tolerate the typical IPR course of three hours of therapy a day for 7-10 days.

The SSFT program was developed at Beaumont Health in Troy, Michigan for individuals with the ultimate desire to DC home who would benefit from further rehabilitation but are unable to tolerate a traditional course of IPR. The patient must be diagnosed with advanced cancer or advanced/end-stage lift-limiting condition, demonstrate home safety concerns that required substantial family/caregiver training, and have social support available to participate in the program and to provide care post-discharge [28]. The patient in the current case report was chosen as a candidate for the SSFT program because of his goal to return home, yet his family was not comfortable caring for him and was seeking further training following his surgery.

Phase 3

The patient was admitted to the hospital’s IPR with an admitting diagnosis of myelopathy of the thoracic region (ICD 10 code G72.9). The initial evaluation in IPR revealed gross R LE MMT graded 2/5 with exception of 0/5 for R DF. The L LE strength was grossly 0/5. The patient was too fatigued to attempt log rolling or transfer to EOB during the IPR evaluation. He was agreeable to transfer to a bedside chair via a mechanical lift with the TLSO donned. He was able to sit up in the chair with back support, bilateral UE support, and minimal physical assistance from the therapist for 30 minutes. Education on pressure relief, repositioning in the chair, and AAROM of bilateral LE were completed. 

During the third phase of physical therapy, the family was educated on mechanical lift operations, including all safety precautions, and completed multiple transfers with therapist feedback.

The patient was progressively tolerating less activity and requiring more rest breaks during each session, thus significant time was allocated to educating the family on pain management including PROM and manual techniques for the extremities. His wife was able to demonstrate competency in performing these techniques independently. Toward the end of this phase, the family was instructed on the home set up, including a ramp into the home, hospital bed, and the amount of physical assistance the patient would require upon DC. The patient’s tolerance to activity was rapidly declining, and at the end of the last treatment day, the patient experienced extreme difficulty conversing. The wife engaged in a discussion regarding hospice philosophy in which the therapist affirmed the value of hospice while providing therapeutic listening and emotional support to the family.

Outcomes

The patient was treated for a total of 13 acute care visits and five IPR visits throughout his 30-day hospitalization. Prior to surgery, the patient required minimal assistance for bed mobility and fluctuated between maximum assistance of one person to moderate assistance of two people for transfers. He was able to complete minimal lateral steps and stand pivot to WC at the bedside with RW. 

During IPR SSFT, the patient slowly declined in functional mobility and required increased physical assistance as demonstrated by dependence on a mechanical lift for all out-of-bed mobility. He was able to tolerate sitting up in a stretcher chair to participate in his daughter’s wedding, which was his personal goal. Following the wedding, the family decided that no further therapy services were warranted secondary to a progressive medical condition; they wished to spend the remaining quality time with the patient. The patient was unable to participate in therapy due to worsening pain, difficulty breathing, and difficulty speaking. Doppler ultrasounds were conducted on the LE and revealed bilateral deep venous thrombii (DVTs).

Functional mobility goals were not met at DC, although he did achieve one of two personal goals as he was able to sit up to attend his daughter’s wedding. The patient did not accomplish his other personal goal of returning home. He was DC off IPR and readmitted to the acute care floor under IP hospice and unfortunately expired within 24 hours. See Table 7 for a synopsis of outcomes for a continuum of care through a hospital stay.

**Table 7:** Summary of Clinical Outcomes EOB: edge of bed; WC: wheelchair; R: right; L:left; AMPAC: Activity Measure for Post Acute Care; RW: rolling walker; s/p: status post; SAR: subacute rehabilitation; IPR: inpatient rehabilitation; DC: discharge

	Supine<>Sit EOB	Transfer Sit<>stand	Transfer Bed<>WC	Strength		AMPAC	DC Recommendation
				R	L		
Inpatient Initial Evaluation (Visit 1)	Maximum assistance	Moderate assistance, with RW	Moderate assistance, with slide board	3/5	2/5	9	Home with physical assist
Inpatient Re-Evaluation S/p Decompressive Laminectomy (Visit 8)	Maximum assistance, 2 person assist	Not assessed	Not assessed	Not assessed	Not assessed	6	Home with physical assist
Visit 10	Maximum assistance, 2 person assist	Not assessed	Not assessed	Not assessed	1/5	7	SAR
Re-evaluation for DC recommendation (Visit 13)	Moderate assistance, 2 person assist to EOB Minimum assistance, 2 person assist to log roll	Not assessed	Not assessed	1/5	0/5	8	IPR Short Family Stay
Inpatient Rehabilitation Evaluation	Maximum assistance, 2 person assist	Not assessed	Dependent, Mechanical lift	2+/5	0/5	Not assessed	Home with physical assist
Discharge from Inpatient Rehabilitation	Maximum assistance, 2 person assist	Not assessed	Dependent, Mechanical lift	Not assessed	Not assessed	Not assessed	Home with hospice

## Discussion

The purpose of this case report was to demonstrate the crucial role of physical therapy throughout the continuum of care in a patient with metastatic rectal cancer with a rapid onset of physical impairments secondary to spinal cord compression.

Traditional rehabilitation focuses on preventing and restoring physical impairments resulting from physical harm, injury, or surgery with the aim to achieve maximal recovery, allowing the patient to return to a prior level of function [14]. With a terminal diagnosis, prior level of function goals may not be attainable, and many individuals receiving palliative and hospice care will not demonstrate functional improvement in objective standardized outcome measures as is expected with traditional rehabilitation. Realizing the progressive decline of end-stage disease, The American Physical Therapy Association made a position statement in 2019 supporting the rights of individuals’ access to physical therapy services in palliative care and hospice, regardless of medical prognosis [29].

Those with end-stage and progressive cancer diagnoses experience hospitalization secondary to shortness of breath, generalized weakness, pain, nausea/vomiting, among others. Lehmann et al. found of those diagnosed with cancer in the hospital, 35% experience functional deficits due to weakness, 32% require assistance with ADLs and 23% experience limitations in ambulation [30]. Despite the anticipated decline in function with hospitalization, 85% of patients in the terminal stage want to be able to walk or move around in a WC [31]. Maintaining the highest level of functional abilities, especially mobility through rehabilitation therapies is one of the most fulfilling goals for those with a terminal disease; however, these services are underutilized despite the growing body of literature supporting the benefits [8].

When individuals are unable to complete out-of-bed mobility, feelings of depression and anxiety may increase and impact the patient’s QoL. Okamura et al. completed a studied the psychological aspects of a terminal diagnosis, finding that rehabilitation is not only effective in addressing physical deficits but also improved emotional and psychological states in both patients and family members that participated in rehabilitation [32]. Similarly, Lowe et al. found a positive correlation between physical activity and QoL in individuals with advanced cancer [33]. The feasibility of rehabilitation is exceptionally attainable as demonstrated via Jensen et al. who found that 93% of patients with terminal cancer were able to perform a physical exercise at least once during the hospital stay [34].

Supportive care was used initially in the presented case. With the effects of disease progression, a quick transition of services to palliative physical therapy started with *rehabilitation in reverse*. As reviewed previously, the patient initially presented to the EC independently with no AD. Within the span of 30 days, the patient would transition from independent ambulation to the use of an RW to stand pivot, followed by slide board transfer, and finally dependence on a mechanical lift for out of bed (OOB) mobility. It is important to note that not all patients experience such rapid onset of impairments and that some may undergo *rehabilitation in reverse* for a much longer time frame.

This case also depicts the effective implementation of *skilled maintenance *therapy as it was a significant focus following elective surgery. *Skilled maintenance *was chosen to support the final goal of sitting up for the patient’s daughter’s wedding. The therapeutic activity was the main emphasis during this phase of rehabilitation to optimize the benefits of upright positioning at EOB. Some therapists may perceive the interventions provided under *skilled maintenance* therapy as unskilled services given the patient’s low tolerance to upright activity, inability to complete OOB mobility, continual dependence for mobility, and minimal to no progress with functional outcome measures. Physical therapists are movement experts, and when family or other health care professionals (nursing) are unable to complete mobilization including transfers safely and independently, the services provided are deemed skilled by physical therapy [12,29]. It is fair to state that without the *skilled maintenance* therapy provided by physical therapists, the patient would not have achieved his goal of sitting up for his daughter’s wedding.

With death being inevitable, Kinoshita et al. found that the quality of death and dying was highest in the home and lowest in the hospital [35]. Quality of death and dying was measured with both physical and psychological components inclusive of symptom relief (pain), time with family, environmental comfort, and being respected as an individual [35]. Gomes et al. also found that dying at home as compared to the hospital was better for pain and grief of loved ones so long as the patient and caregiver agree on the location (home vs hospital), home visits were covered by palliative care, and relatives/caregivers were given time off of work [36]. His family underwent extensive family training on transfers and safe patient handling prior to surgery, but medical instability and uncontrolled pain following surgery were substantial barriers for the family to feel confident completing mobility independently at home. IPR SSFT was initiated, but unfortunately, the patient did not tolerate this episode of care and was appropriate for IP hospice. Although the patient did not achieve DC home, the role of physical therapy, in this case, provided the patient an increased QoL during his remaining days.

Limitations

A major limitation to this case report was the lack of consistent outcome measures secondary to changes in physical performance. The 5STS utilized on initial evaluation was not safe nor appropriate to retest at further points in care. The modified sit and reach test was utilized at one random visit throughout the episode of care, but there was no carry-over or follow-up at subsequent visits. The AMPAC was the only outcome measure utilized consistently throughout the hospital stay but was not sensitive to the slight changes in mobility as the patient became non-ambulatory. This case report demonstrates the need for validated and reliable functional outcome measures for non-ambulatory patients with terminal and progressive diagnoses. Lower level valid and reliable functional outcome measures are required to accurately assess patient status and mobility, specifically related to a progressive, terminal disease such as the diagnosis presented in the current case.

## Conclusions

In summary, physical therapy plays an integral role in those with terminal cancer. Many patients hospitalized with terminal cancer experience a decline in mobility and functional independence, although the majority continue to desire to remain ambulatory and mobile even in the terminal stage. Despite a terminal diagnosis and progressive physical impairments, the patient in the current case desired to maintain optimal levels of independence, allowing for participation in his daughter’s wedding. Rehabilitation services assist in improving functional mobility, independence, caregiver confidence, and the patient’s sense of control aiming to improve QoL. Supportive and *skilled maintenance* physical therapy services allow for a patient to achieve final personal goals, which may not be achieved without the expertise of rehabilitation professionals.
